# Circular RNA circFLNA inhibits the development of bladder carcinoma through microRNA miR-216a-3p/BTG2 axis

**DOI:** 10.1080/21655979.2021.2008659

**Published:** 2021-12-02

**Authors:** Shuangquan Lin, Lei Wang, Zimin Shi, Anyi Zhu, Gan Zhang, Zhengdong Hong, Cheng Cheng

**Affiliations:** a Department of Urology, The Second Affiliated Hospital of Nanchang University, Nanchang, Jiangxi, China; b College of Pharmacy, Nanchang Medical College, Jiangxi, China

**Keywords:** CircFLNA, miR-216a-3p, BTG2, bladder carcinoma, stem cells, malignant phenotype

## Abstract

Recent studies have shown that circular RNA circFLNA is abnormally expressed in a variety of malignant tumors, but its role and mechanism in bladder carcinoma (BCa) are still unclear. The present paper aims to contribute to research on the effects and mechanism of circFLNA on the malignant phenotype of BCa. In this study, the expressions of circFLNA, miR-216a-3p and BTG2 in BCa and BCa cells (EJ, T24, 5637, TCC-SUP) were detected by qRT-PCR. EdU staining, colony formation, Transwell assay, wound healing assays, and sphere formation assay were used to measure the cell proliferation, viability, invasion, migration, and cell stemness of BCa cells after circFLNA overexpression. In addition, the correlation existed between miR-216a-3p and circFLNA or BTG2 was confirmed by Dual-Luciferase Reporter assay and RNA pull-down. Western blot was utilized to determine the expression of BTG2, MMP2, epithelial-mesenchymal transition (EMT)-related proteins (vimentin, E-cadherin) and stem cell-specific proteins (CD34, OCT4, SOX2). Our study confirmed that downregulated circFLNA and BTG2 expression and upregulated miR-216a-3p were found in both BCa tissues and cell lines. Meanwhile, upregulated circFLNA inhibited proliferation, invasion and migration, EMT and stemness of BCa cells. *MiR-216a-3p* was a target gene of circFLNA and could target BTG2. Further analysis finally demonstrated that circFLNA sponged miR-216a-3p and indirectly promoted BTG2 expression, ultimately regulating proliferation, migration, invasion and EMT of BCa cells. In conclusion, circFLNA inhibits the malignant phenotype of BCa cells and their stemness through miR-216a-3p/BTG2, thus suppressing BCa progression.

## Introduction

Bladder carcinoma (BCa) is currently described as the most common malignancy in the urinary system, and as a malignancy with the ninth highest prevalence in the world [1,2]. Among new tumor cases, the incidence of BCa was 7% in men and 2% in women, with 65 years as the average age of onset. Approximately 75% of new BCa cases with tumor confined to the bladder, while 25% present with regional lymph node metastasis or distant metastasis [3]. Surgery, radiotherapy, chemotherapy, and biological therapy are still considered to be the main types of treatment for BCa, and transurethral resection of bladder tumor and chemotherapy are most frequently employed. But these treatments also have some limitations, for example, a recurrence rate of about 70% in patients who undergo transurethral resection of bladder tumor [4–6]. Therefore, for the treatment of BCa, it is particularly vital to find highly sensitive diagnostic and prognostic targets.

Circular RNAs (circRNAs) are named for their circular structure forming by covalently binding, which is critical in regulating tumor development. CircRNA promises to be a potential biomarker for disease diagnosis and treatment due to its advantages of high stability, tissue- and disease-specificity. CircRNA100146 controls RNF2 by sponging miR-149-5p and consequently promotes BCa, suggesting its possibility as a novel biomarker for BCa [7]. CircACVR2A, with downregulated expression in BCa tissues and cells, contributes to the inhibition of BCa celll proliferation, invasion, and migration, and this circRNA can regulate EYA4 expression by sponging miR-626 [8]. Circ0092012 is formed by the cyclization of exons 9–15 of the filamin A (FLNA) gene, also known as circFLNA. CircFLNA, showing dysregulated expression in a variety of malignancies, is associated with poor prognosis of tumors [9]. For example, circFLNA presents with upregulated expression in gastric cancer, and the circFLNA/miR-646/PFKFB2 axis is a potential therapeutic target for gastric cancer [10]. CircFLNA contributes to a decrease in FLNA expression through sponging miR-486-3, which provides a strategy targeting circFLNA for treating invasive laryngeal squamous cell carcinoma. However, it is still unknown about the expression and function of circFLNA in BCa. In the current study, we hypothesized that circFLNA plays an important role in the progression of BCa. We first examined circFLNA expression in BCa tissues and cell lines, and its mechanism in BCa cell growth, migration and invasion. This study aimed to reveal the biological functions and potential molecular mechanisms of circFLNA in BCa progression, which provided new clues for BCa clinical diagnosis and treatment.

## Materials and methods

### Patient tissue

The first step was harvesting surgically resected BCa tissue (BCa) and normal tissue (Normal) from 21 BCa patients between 2017 and 2018 in the Department of Urology of our hospital. Tissues confirmed as BCa by pathological examination were included as BCa tissues, but the metastatic BCa specimens were excluded. Each patient was informed about the purpose and significance of collecting clinical specimens and signed informed consent. This study had obtained approval from the Ethics Committee of the Second Affiliated Hospital of Nanchang University (No. Review [2018] No. (090).

### Cell culture

BCa cell lines (EJ, T24, 5637, TCC-SUP) and human normal bladder epithelial cells (SV-HUC-1) were purchased from Shanghai Institutes for Biological Science, Chinese Academy of Sciences. SV-HUC-1 cells were cultured in F-12 K medium, T24 cells in McCoy’s 5A medium, and EJ and 5637 cells in 1640 medium, and TCC-SUP in MEM medium. All media with a supplement of 1% penicillin-streptomycin and 10% Fetal bovine serum (FBS; Invitrogen, Carlsbad, CA, USA) were prepared, followed by placing them in an incubator with 5% CO_2_ and 37 °C.

### qRT-PCR

Following the extraction of total RNA from cells and tissue using TRIZOL (Invitrogen, Carlsbad, CA, USA), reverse transcription was performed according to the instruction of a reverse transcription kit (TaKaRa, Tokyo, Japan). Then, gene quantification on the Light Cycler480 system (Roche, Indianapolis, IN, USA) was carried out under the instruction of the PCR kit (SYBR Green Mix, Roche Diagnostics, Indianapolis, IN). Specific thermal cycling parameters were listed as follows: 95°C for 10 s; 45 cycles of 95°C for 5 s, 60°C for 10 s, and 72°C for 10 s; finally, 5 min extension at 72°C. Each reaction of qRT-PCR set three replicates. Quantitative analysis using 2^−ΔΔCt^ method was conducted with GAPDH and U6 as normalization controls for circRNA/mRNA and miRNA, respectively. The primer sequence of each gene is listed in Table 1.

**Table 1.** qRT-PCR primer sequences

Primer		Sequence (5ʹ–3ʹ)
circFLNA	Forward	CAACAAGTTCACTGTGGAGACCA
	Reverse	TGTAGGTGCCAGCCTCATAAGG
BTG2	Forward	GCAGAGGCTTAAGGTCTTCAGC
	Reverse	TGGTTGATGCGAATGCAGCGGT
GAPDH	Forward	GGAGCGAGATCCCTCCAAAAT
	Reverse	GGCTGTTGTCATACTTCTCATGG
miR-216a-3p	Forward	CTCAGCTGGCAACTGTG
	Reverse	GAACATGTCTGCGTATCTC
U6	Forward	UUCUCCGAACGUGUCACGUTT
	Reverse	UGACACGUUCGGAGAATT

### Cell transfection

GenePharma (Shanghai, China) provided miR-216a-3p mimics (miR-216a-3p) and its negative control (NC mimic) (100 nM), miR-216a-3p inhibitor (in-miR-216a-3p) and its negative control (NC inhibitor) (100 nM), lentiviral vector of circFLNA (circFLNA) and its negative control (vector), siRNA-circFLNA and its negative control (siRNA). After cells reaching 70–80% confluence in 6-well plates, they were transfected with the above plasmids and vectors, respectively, under the instruction of Lipofectamine 2000 (Invitrogen, Carlsbad, CA, USA). After 6 h of transfection, another 48 h cell culture was carried out in fresh media.

### EdU staining

On completion of 48 h culture of transfected cells in 96-well plates (5000 cells/well), EdU solution was added to the plates at a ratio of 1:2000 to the media for 2 h cell culture. After EdU labeling and aspiration of the solution, a three-time PBS rinsing was carried out (3–5 min/time). Then, 4% paraformaldehyde (200 μl/well) was utilized for 15 min fixation at ambient temperature, followed by an addition of 2 mg/mL glycine (200 μl/well). Subsequently, after 5 min incubation in a decolorization shaker, the glycine solution was aspirated. Subsequently, 200 μl PBS was added to each well, followed by 5 min PBS washing in the decolorization shaker. On completion of washing step, PBS was aspirated, and the cells were incubated with TritonX-100 for 15 min and then rinsed with PBS for a period of 5 min. The next step was to mix 430 μl of Click Reaction Buffer, 420 μl of CuSO, 4881 μl of Azide, and 50 μl of Click Additive Solution and add the mixture into 96-well plates (200 μl/well) for 30 min incubation in the dark. For nuclear staining, each well was added with 200 μl 1000X Hoechst 33,342 diluted with PBS (1:1000), followed by 10 min incubation avoiding light at the ambient temperature. The final step was to rinse the cells three times (3–5 min/time). Under a fluorescence microscope, proliferating cells (green) and nuclei (blue) were observed.

### Colony formation assay

After cell counting by a hemocytometer, the cells were seeded in 12-well plates (density controlled at 800 cells/well, and 3 replicate wells for each group), followed by placing them in an incubator for continued culture. During the culture period, the culture medium was changed into a fresh one every 2–3 days. And from the seventh day after seeding to the 12-well plates, the culture medium was aspirated before cell colonies (number of cells ≥50) fused with each other, followed by PBS washing for two times. Finally, 20 min staining using crystal violet and rinsing with PBS were performed, then the stained cells were observed and photographed. The number of cell colonies formed in each group was calculated.

### Transwell assay

Matrigel was removed from −20°C and placed in the refrigerator at 4°C overnight in advance to allow it to melt into liquid. The bottom membrane of the transwell insert was coated with diluted Matrigel (Matrigel:medium = 1:8). A sufficient number of cells in good growth condition were collected and diluted to appropriate concentrations with a serum-free medium. The aseptic Transwell inserts were placed in a 24-well plate and then placed into an incubator for a period of 18 h, with the lower chamber prepared by adding 800 μl of complete culture medium containing 10% FBS and the upper chamber by adding 100 μl of cells with equal concentration. Then, the transwell inserts were gently washed three times with PBS prior to 30 min cell fixation using 4% paraformaldehyde. After fixation, the transwell inserts were taken out carefully. A cotton swab was utilized to remove the non-invaded cells in the upper chamber by gently wiping, followed by three PBS washing steps for the inserts. The lower side of the insert membrane was soaked in 0.5% crystal violet for 20 min staining, followed by a three-time ddH2O washing for the inserts. Finally, the inserts were dried at ambient temperature, and five randomly selected fields were photographed. By using ImageJ, the number of cells in each field was statistically analyzed.

### Wound healing assay

Sufficient transfected cells with good growth status were evenly covered in a 6-well plate to ensure the same number of cells in each well. After cells reaching 100% confluence, a sterilized 100 μl tip perpendicular to the plate was utilized to make a scratch in the middle of each well, and cells scattered from the scratches were removed with PBS. Then, serum-free medium was added in each well for cell culture in an incubator with 5% CO_2_ and 37 ℃. Finally, the samples were photographed, labeled and recorded. Specifically, the scratches were observed and photographed under a microscope at 0 and 18 h, respectively, and their change in the width was recorded and subjected to statistical analysis.

### Western blot

On completion of cell lysis using RIPA buffer (Beyotime Biotechnology, Shanghai, China), the obtained protein samples were detected to confirm protein concentration using BCA kit (Beyotime Biotechnology) and corresponding volume of them was taken to mix with sample loading buffer (Beyotime Biotechnology). Then, protein denaturation was achieved by 5 min heating in a boiling water bath. After protein sample preparation, the next step was to separate proteins by SDS-PAGE and to transfer them onto a PVDF membrane. On completion of 1 h blocking step with 5% skimmed milk at ambient temperature, primary antibodies GAPDH (5174S), MMP2 (40994S), E-cadherin (14472S), vimentin (5741SA), CD44 (37259S), OCT4 (2750S), SOX2 (3579S), BTG2 (ab197362, Abcam, Boston, USA) served to overnight co-incubation with the proteins at 4°C in a shaker. All primary antibodies were diluted as 1:1000, and except primary antibody BTG2, the others were provided by Cell Signaling, Boston, USA. The next day, after three rinsing steps (10 min/time), the proteins were incubated with secondary antibody (horseradish peroxidase (HRP)-labeled goat anti-rabbit IgG, 1:5000, CoWin Biosciences, Beijing, China) for 1 h at ambient temperature, followed by three washing steps again (10 min/time). After dropping the developer on the membrane, the detection was completed using a chemiluminescence imaging system (Bio-Rad).

### Sphere formation assay

The special medium for sphere formation (a system of 30 mL) was as follows: 29.4 mL DMEM-F12 + 60 μL 50xB27 + 3 μL LEGF (200 μg/mL) + 12 μL bFGF (20 ng/mL). When BCa cell attachment reaching 80%, the cells were digested, centrifuged, and washed with PBS for 2 times, resuspended with special medium. After that, in 96-well plates, each well contained 1000 resuspended cells was supplemented with 200 μL of medium. Fresh medium was added every two days. Ten days later, the sphere was photographed with AxioVision software for data analysis.

### Extraction of nucleus and cytoplasm

The transfected cells were collected and processed under to the instruction of a nuclear and cytoplasmic RNA extraction kit (Amyjet, China). Cells were lysed by pre-colded lysis buffer J and centrifugated. Subsequently, the supernatant containing cytoplasmic RNA was transferred. Buffer SK solution was added to the supernatant and pellet, respectively, followed by vortex, and then 96–100% alcohol was added for vortex again. Next, centrifugation (6000 rpm, 1 min) was performed and the resulting supernatant was aspirated. Wash solution was adopted for washing the spin column, and elution buffer for eluting the RNA.

### Dual-luciferase gene reporter assay

The online databases ENCORI (http://starbase.sysu.edu.cn/) and TargetScan (http://www.targetscan.org/vert_72/) were used for predicting the binding site. Wild-type (WT) and mutant (MUT) sequences (circFLNA-WT, circFLNA-MUT, BTG2-WT, BTG2-MUT) of the binding site were designed and synthesized, followed by inserting the sequences into the luciferase reporter vector (pGL3-Basic) respectively. Subsequently, the sequences were co-transfected into HEK293T cells with miR-216a-3p mimics or mimics NC, respectively. For complete cell lysis, 100 µl of cell lysis buffer was added to the transfected cells and then the samples were placed in a shaker for a period of 20 min at ambient temperature. Next, 50 µl of luciferase reaction solution and 50 µl of lysed cell suspension was mixed for measuring Firefly luciferase activity, and then mixed with 50 µl of Stop&Glo reagent (Promega, USA) for confirming Renilla luciferase activity. With Renilla luciferase activity as an internal reference, the relative activity of luciferase (Firefly:Renilla) was calculated. Three replicates were set up for the experiment.

### RNA pull-down

The experiment was performed as described previously [11]. In this experiment, 1 × 10^7^ circFLNA-overexpressing 5637 cells were fixed at 37°C for 10 min, then lysed and sonicated. After centrifugation, the supernatant was incubated with biotin-labeled circFLNA probes or negative control (Sangon Biotech, China) for 4 h at 25°C, followed by incubation with streptavidin magnetic beads overnight at 4°C. The next day, after five washes with RIP buffer, TRIzol was added for RNA extraction. The recovered RNA was detected and analyzed by qRT-PCR.

### Statistical analysis

By utilizing SPSS 22.0, statistical analysis of obtained experimental data was achieved, with mean ± standard deviation (SD) to express the data. *T*-test was adopted for comparison between two groups, one-way analysis of variance for comparison among groups, and Turkey method for multiple comparisons of means. Pearson’s correlation analysis was adopted for confirming the correlation between miR-216a-3p and circFLNA or BTG2 in BCa tissues. All images were plotted using GraphPad Prism8.0. The cutoff value indicating a significant difference was *P* < 0.05.

## Results

### Downregulated expression of circFLNA in bladder carcinoma tissues and cells

**Figure 1.**
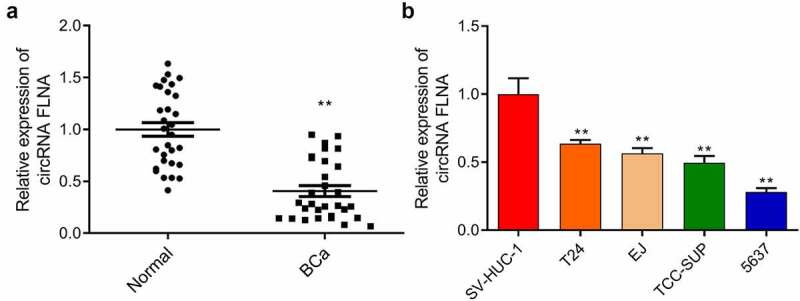
Downregulation of circFLNA expression in bladder carcinoma tissues and cells

First, according to the qRT-PCR result on circFLNA expression in BCa tissue, a lower circFLNA expression was identified in BCa tissue compared to normal tissue (Figure 1a). Then, according to the qRT-PCR result on its expression in BCa cells, circFLNA expression was significantly lower in T24, EJ, TCC-SUP, and 5637 cells than that in SV-HUC-1, with the lowest expression in 5637 cells (Figure 1b). Collectively, circFLNA expression might be related to BCa occurrence.

- qRT-PCR-based measurement of circFLNA expression in Normal (normal tissues) and BCa (bladder carcinoma tissues) groups, **P < 0.01 vs. Normal group; (b) qRT-PCR-based measurement of circFLNA expression in human bladder epithelial cells (SV-HUC-1) and bladder carcinoma cells (T24, EJ, 5637, TCC-SUP); **P < 0.01 *vs*. SV-HUC-1 group.

### Inhibition of malignant development of bladder carcinoma cells by circFLNA

**Figure 2.**
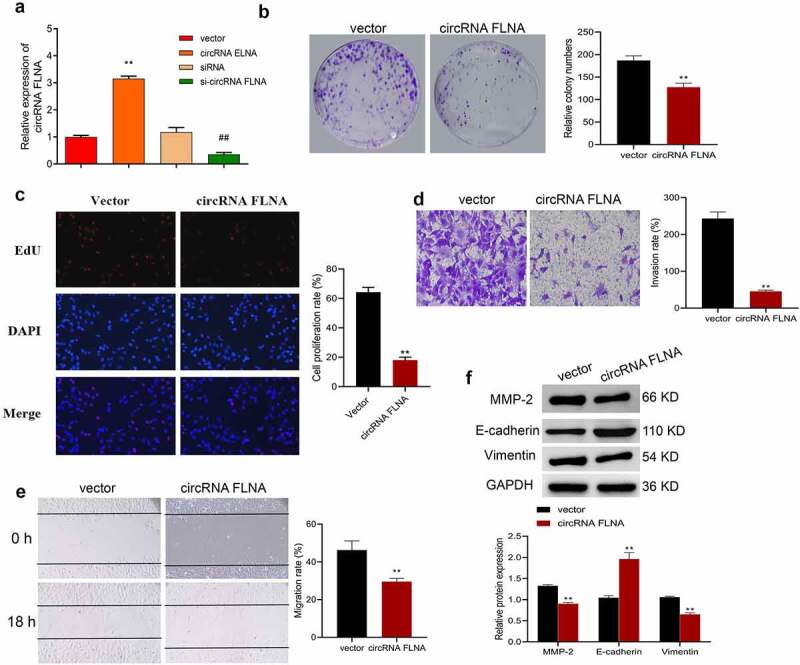
Effect of circFLNA on the proliferation, invasion, and migration of bladder carcinoma cells

Further investigation of biological function of circFLNA was achieved by detecting proliferation, cell viability, invasion and migration of BCa cells, and expression of related proteins of epithelial-mesenchymal transition (EMT). In 5637 cells, upregulation and downregulation of circFLNA was successfully achieved by overexpression and silencing of circFLNA, respectively (Figure 2a-b). In 5637 cells, overexpression of circFLNA inhibited cell viability, proliferation, invasion, and migration (Figure 2c-e) significantly decreased MMP-2 and vimentin expression while increased E-cadherin expression (figure 2f). Taken together, circFLNA contributed to an inhibition of BCa malignant development.

- qRT-PCR-based measurement of circFLNA expression in 5637 cells; (b–f) In 5637 cells, cell viability was confirmed by colony formation assay (b), proliferation by EdU (c), invasion by Transwell assay (d), migration by wound healing assay (e), and expression of EMT-related proteins by Western blot (f); **P < 0.01 *vs*. Vector group, ^##^*P* < 0.01 *vs*. siRNA group.

### CircFLNA inhibits the stemness of bladder carcinoma cells

**Figure 3.**
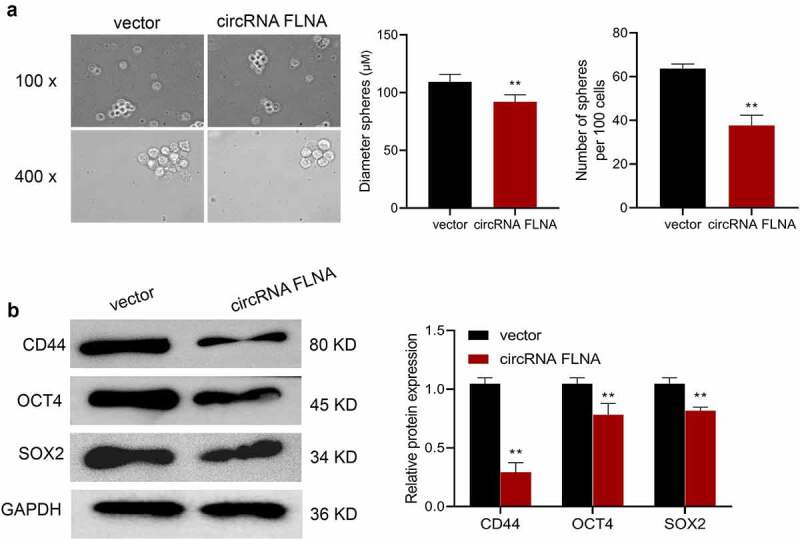
Effect of circFLNA on bladder carcinoma cell stemness

Cancer stem cells (CSCs) are currently considered to be the center of malignant transformation, growth and metastasis of cancer cells [12,13], and increasing novel anticancer drugs target CSCs. Sphere formation assay was therefore carried out and confirmed the inhibition of stemness of BCa cells by circFLNA. Specifically, overexpression of circFLNA could significantly reduce the sphere-forming efficiency, and the number and diameter of spherical pellets in BCa cells (Figure 3a). Further, according to Western blot results, in BCa cells, overexpression of circFLNA led to a significant decrease in the expression of stem cell markers, CD44, OCT4, and SOX2 (Figure 3b). Collectively, upregulated expression of circFLNA was associated with an inhibition of BCa cell stemness.

- Sphere formation assay for detecting the effect of upregulated circFLNA expression on sphere-forming efficiency, number and diameter of spherical pellets in 5637 cells;

- (B) Western blot for detecting the effect of upregulated circFLNA expression on stem cell markers in 5637 cells; **P < 0.01 *vs*. Vector group.

### CircFLNA adsorbed miR-216a-3p in bladder carcinoma cells

**Figure 4.**
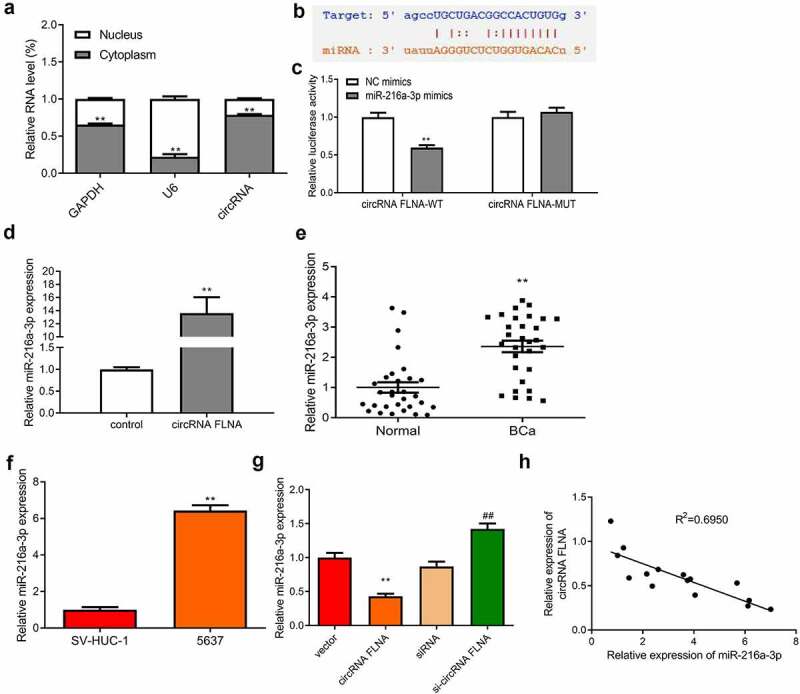
Association between circFLNA and miR-216a-3p expression

The mechanism by which circFLNA affected BCa was then investigated. Expression distribution of circFLNA in the nucleus and cytoplasm of 5637 cells was first examined. CircFLNA expression was higher in the cytoplasm relative to the nucleus, indicating that this circRNA was mainly localized in the former (Figure 4a). This meant that circRNA had the potential to become an endogenous competitor RNA to bind to microRNAs and affect downstream target genes competitively. ENCORI database was used to search the possible miRNA sponged by circFLNA, and the potential binding site was found between circFLNA and miR-216a-3p (Figure 4b). Further dual-luciferase gene reporter assay was carried out to validate the binding. It was confirmed that overexpression of miR-216a-3p caused no effect of luciferase activity of circFLNA-MUT vector, but markedly suppressed that of circFLNA-WT vector (Figure 4c). RNA pull-down assay also demonstrated that in 5637 cells, circFLNA precipitated miR-216a-3p (Figure 4d).

Upregulated miR-216a-3p expression was found in both BCa tissue and cells (Figure 4e-f). Overexpression of circFLNA allowed miR-216a-3p to be reduced, while downregulated circFLNA significantly increased miR-216a-3p expression (Figure 4g). As determined by Pearson’s correlation analysis, circFLNA and miR-216a-3p had a negative correlation (Figure 4h). The above results suggested that circFLNA adsorbed miR-216a-3p and negatively regulated its expression in BCa cells.

- qRT-PCR-based measurement of circFLNA expression in the nuclear and cytoplasm of 5637 cells, **P < 0.01 *vs*. Nucleus group; (b) Online database ENCORI was used to predict the binding site between circFLNA and miR-216a-3p; (c) Determination of miR-216a-3p targeting circFLNA by dual-luciferase gene reporter assay; (d) Confirmation of interaction between circFLNA and miR-216a-3p by RNA pull-down assay; (e, f) qRT-PCR-based measurement of miR-216a-3p expression in tissues (e, **P < 0.01 *vs*. Normal) and 5637 cells (f, **P < 0.01 *vs*. SV-HUC-1); (g) qRT-PCR-based measurement of miR-216a-3p in 5637 cells in each transfection group, **P < 0.01 *vs*. Vector group, ^##^*P* < 0.01 *vs*. siRNA group; (h) Correlation analysis of miR-216a-3p and circFLNA.

### MiR-216a-3p reverses circFLNA-caused inhibition of malignant development of bladder carcinoma cells

**Figure 5.**
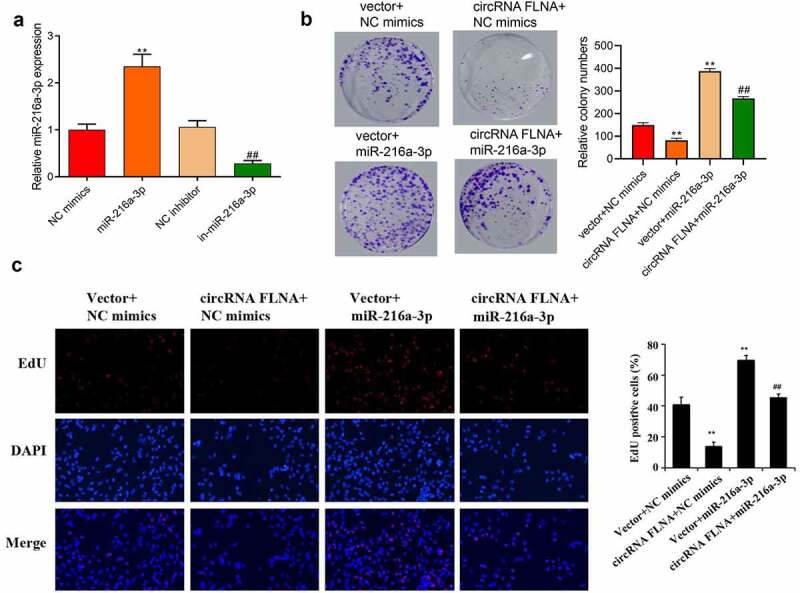
MiR-216a-3p affects inhibitory effect of circFLNA on cell viability and proliferation (a) qRT-PCR-based detection of miR-216a-3p expression in 5637 cells, **P < 0.01 *vs*. NC mimics group, ^##^*P* < 0.01 *vs*. NC inhibitor group; (b, c) 5637 cell viability was detected by colony formation assay (b), and proliferation by EdU assay (c), **P < 0.01 *vs*. Vector + NC mimics group, *^##^P* < 0.01 *vs*. circFLNA + NC mimics group

To further probe the interaction between circFLNA and miR-216a-3p, we observed the effect of circFLNA on BCa cells after overexpression of miR-216a-3p. Transfection of miR-216a-3p mimics led to an increase of miR-216a-3p, while miR-216a-3p inhibitor (in-miR-216a-3p) caused an opposite result (Figure 5a), indicating successful transfection.

**Figure 6.**
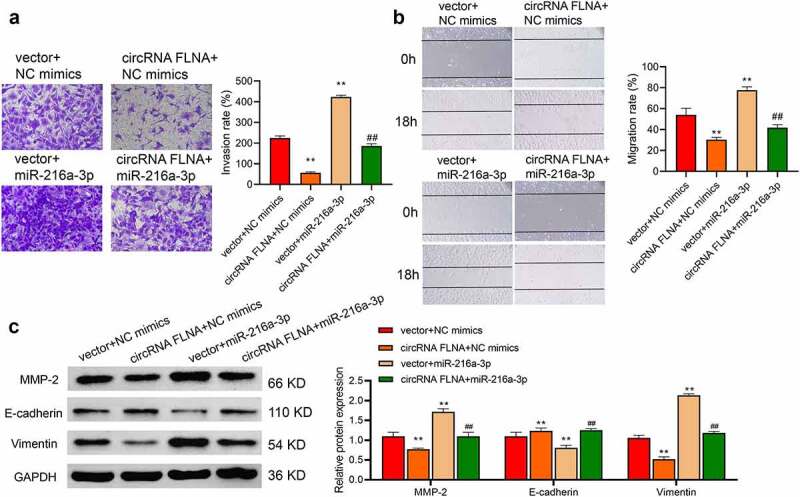
MiR-216a-3p affects inhibitory effect of circFLNA on cell invasion and migration

Compared with the circFLNA+ NC mimics group, overexpression of miR-216a-3p caused increases of cell viability (Figure 5b), proliferation ability (Figure 5c), invasion (Figure 6a), and migration ability (Figure 6b). And overexpression of miR-216a-3p also reversed the inhibitory effect of circFLNA on MMP-2 and vimentin protein expression and promoted E-cadherin expression (Figure 6c). It showed that circFLNA could exert its anticancer effect by inhibiting miR-216a-3p.

- C) In 5637 cells, invasion was detected by Transwell assay (a), migration by wound healing assay (b), and MMP-2, E-cadherin, and vimentin expression by Western blot, **P < 0.01 *vs*. Vector + NC mimics group (c), ^##^*P* < 0.01 *vs*. circFLNA + NC mimics group.

### MiR-216a-3p reverses circFLNA-caused inhibition of stemness of bladder carcinoma cancer cells

**Figure 7.**
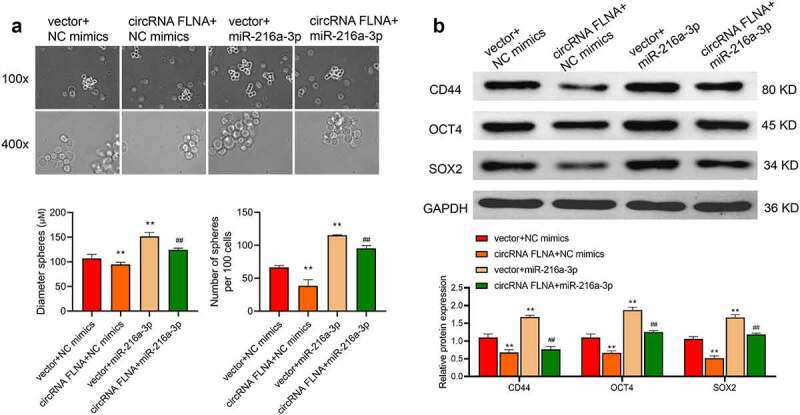
Effect of miR-216a-3p on circFLNA-caused inhibition of the stemness of bladder carcinoma cells

By sphere formation assay, we investigated the effect of miR-216a-3p on circFLNA-caused inhibition of stem cell marker-related proteins. The results showed that miR-216a-3p reversed the inhibitory effect of circFLNA on sphere-forming efficiency, and number and diameter of spherical pellets (Figure 7a), and reversed circFLNA-caused inhibition of stem cell markers, CD44, OCT4, and SOX2 (Figure 7b). Taken together, miR-216a-3p reversed circFLNA-caused inhibition of the stemness of BCa cells.

- Stem cell sphere-forming assay for detecting the number and diameter of spherical pellets in 5637 cells; (B) Western blot for assessing expression of stem-related marker proteins in 5637 cells; **P < 0.01 *vs*. Vector + NC mimics group, ^##^*P* < 0.01 *vs*. circFLNA + NC mimics group.

### CircFLNA regulates BTG2 expression by sponging miR-216a-3p in BCa

**Figure 8.**
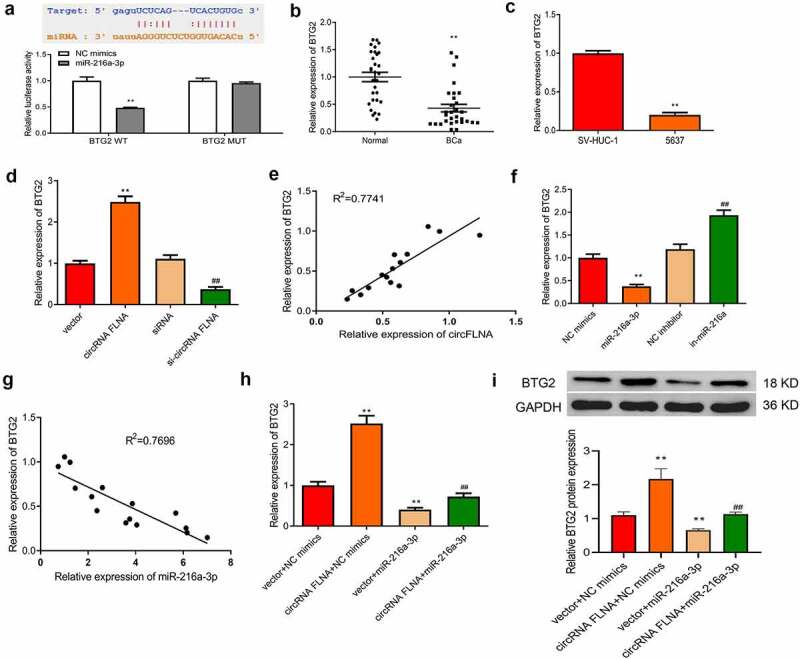
Association among circFLNA, miR-216a-3p, and BTG2

Target genes affected by the regulation of miR-216a-3p by circFLNA were further studied. The downstream targets of miR-216a-3p were predicted by the online database TargetScan (http://www.targetscan.org/vert_72/). As shown in Figure 8a, BTG2 was one target of miR-216a-3p. The dual-luciferase gene reporter assay further confirmed that overexpression of miR-216a-3p caused no effect of luciferase activity of BTG2-MUT vector, but markedly suppressed that of BTG2-WT vector (Figure 8a), suggesting that miR-216a-3p targeted BTG2. It was also found that BTG2 expression was markedly decreased in BCa tissues and cells (Figure 8b, c).

Overexpression of circFLNA resulted in an increase of BTG2 expression, while silencing of circFLNA led to an opposite result (Figure 8d). Pearson’s correlation analysis determined that circFLNA and BTG2 had a positive correlation (Figure 8e).

Additionally, overexpression of miR-216a-3p caused a reduction of BTG2 expression, while silencing of miR-216a-3p led to an opposite result (figure 8f). According to Pearson’s correlation analysis, miR-216a-3p and BTG2 had a negative correlation (Figure 8g). In BCa cells, upregulation of BTG2 mRNA and protein expression by overexpression of circFLNA could be reversed by miR-216a-3p (Figure 8h-i). Collectively, circFLNA could regulate BTG2 expression through miR-216a-3p.

- Predication of the binding site of miR-216a-3p to BTG2 by online database TargetScan, and determination of miR-216a-3p targeting BTG2 by dual-luciferase gene reporter assay, **P < 0.01 *vs*. NC mimics group; (b, c) qRT-PCR-based measurement of miR-216a-3p expression in tissues (b, **P < 0.01 *vs*. Normal) and 5637 cells (c, **P < 0.01 *vs*. SV-HUC-1); (d) qRT-PCR-based measurement of BTG2 in 5637 cells in each transfection group, **P < 0.01 *vs*. Vector group, ^##^*P* < 0.01 *vs*. siRNA group; (e) Correlation analysis of circFLNA and BTG2. (f) qRT-PCR for detecting the effect of upregulation or downregulation of miR-216a-3p on BTG2 expression in 5637 cells, **P < 0.01 *vs*. NC mimics group, ^##^*P* < 0.01 *vs*. NC inhibitor group; (g) Correlation analysis of miR-216a-3p and BTG2; (h) qRT-PCR for detecting what effects miR-216a-3p and circFLNA have on BTG2 in 5637 cells; (i) Western blot for evaluating what effects of miR-216a-3p and circFLNA have on BTG2 protein expression in 5637 cells, **P < 0.01 *vs*. Vector + NC mimics group, ^##^*P* < 0.01 *vs*. circFLNA + NC mimics group.

## Discussion

BCa, a common malignancy involving the urinary system, shows high morbidity and mortality. In China, its mortality and incidence rank first among the urinary system tumors [14], and the incidence is still increasing yearly and there is a trend of younger patients [15,16]. Effective diagnosis and treatment of BCa are therefore crucial. With the advent of next-generation sequencing technologies, a large number of circRNAs, in various animal genomes, have been identified, many of which showing high stability and abundant expression. The tissue-specificity of circRNAs contributes to their close relationship with the development of numerous diseases. CircRNAs are considered promising biomarkers and therapeutic targets because of their sequence conservation, biostability, and tissue-specificity [17,18]. Among them, *circFLNA* is a newly discovered gene [19], and some studies revealed that overexpression of circFLNA, XRCC1, and CYP1A1 can promote survival and malignant development of cancer cells [20]. However, what effects circFLNA have on BCa remains to be determined, and given this uncertainty, this study regarding this field has carried out. CircFLNA showed downregulated expression in both BCa tissues and cells, and the upregulated expression caused suppression of BCa cell proliferation, migration, and invasion, and consequently inhibition of malignant expression of BCa. Interference or overexpression of circFLNA could significantly affect the malignant development of BCa cells. It has been reported in the relevant literature that circRNAs achieve the regulation of gene expression through sponging miRNAs and interacting with RNA binding proteins. *MiR-216a-3p* was also found to be a target gene of circFLNA in this study and was of a negative correlation with circFLNA; overexpression of miR-216a-3p reversed circFLNA-caused inhibition of malignant development of BCa cells.

EMT refers to a phenomenon that epithelial cells lose their original cell polarity and their tight junctions and transform toward mesenchyme cells [21]. Increased expression of vimentin and decreased expression of cell adhesion molecules (such as E-cadherin) occur during EMT. Matrix metalloproteinases (MMPs) are a large family of metal-dependent endopeptidases with calcium and zinc ions as cofactors [22] and can mediate EMT to increase tumor invasion and metastasis. It has been shown that miR-34a/LGR4 targets MMP2 to mediate EMT, thus regulating migration and invasion of uveal melanoma cells [23]. Hence, this study used E-cadherin, MMP2, and vimentin proteins as indicators of cell invasion and metastasis and EMT. Upregulation of circFLNA expression resulted in decreases in MMP2 and vimentin but an increase in E-cadherin; miR-216a-3p reversed these effects of circFLNA. These data illustrate that circFLNA/miR-216a-3p can affect EMT, ultimately leading to changes in the malignant phenotype of BCa.

CSCs recently have been found to exist in a variety of malignant tumors, including breast, lung, prostate cancers, and BCa [24]. CSCs possess abilities of continuous self-renewal and continuous proliferation and differentiation [25], and it is critical in cancer occurrence, progression, metastasis, recurrence and resistance to chemotherapy [26]. Commonly used molecular markers to identify CSCs of BCa include CD44, OCT4, ALDH1A1, CK5, and CK14 [27], and abnormally high expression of these molecular markers in BCa tissues often predicts a poor prognosis [28]. Additionally, Fengyu Zhu et al. found that Sox2 is also a marker of CSCs of BCa, suggesting its possibility as a clinical target for BCa therapy [29]. Hence this study used CD44, OCT4, and SOX2 proteins as indicators of stemness of BCa cells. Upregulation of circFLNA inhibited these proteins and affected the stemness of BCa cells; miR-216a-3p reversed these effects of circFLNA.

In a variety of malignancies, *BTG2*, the first identified gene belonging to TOB/BTG gene family [30], acts as an antioncogene to involve in DNA damage repair, apoptosis, proliferation, and cell cycle [31], and shows downregulated expression [32]. For example, BTG2 is downregulated in BCa and is associated with poor prognosis [33]. It has been indicated that BTG2 expression has an epigenetic regulatory role in BCa and is a promising epigenetic target for preventing muscle invasive BCa [34]. In this study, downregulation of BTG2 expression was also found to correlate with a disorder of circFLNA/miR-216a-3p expression in the upstream.

## Conclusion

In summary, this study concludes for the first time that circFLNA is downregulated in BCa and inhibits the malignant development and stemness of BCa cells. CircFLNA promotes BTG2 expression through sponging miR-216a-3p, ultimately achieving the inhibition. Therefore, circFLNA/miR-216a-3p/BTG2 can be used as a BCa biomarker and a potential therapeutic target, providing a new idea for diagnosing and treating BCa in its early stage. However, the effect of circFLNA on BCa progression *in vivo* and its relevance to clinicopathology requires further study.

## Data Availability

The original contributions presented in the study are included in the article/supplementary material. Further inquiries can be directed to the corresponding author.

## Highlights

1. CircFLNA and BTG2 expression was downregulated in BCa, while miR-216a-3p expression was upregulated.
2. Upregulated circFLNA inhibited proliferation, invasion and migration, EMT and stemness of BCa cells.
3. CircFLNA sponged miR-216a-3p and indirectly promoted BTG2 expression.

## References

[1] Antoni S, Ferlay J, Soerjomataram I, et al. Bladder cancer incidence and mortality: a global overview and recent trends. Eur Urol. 2017;71(1):96–108. 27370177 10.1016/j.eururo.2016.06.010

[2] Fu DXX, Wei Z, Yi X, et al. NRP-1 silencing suppresses bladder cancer cell line proliferation both in vitro and in vivo. Anal Quant Cytopathol Histopathol. 2018;40:277–283.

[3] Bladder cancer: diagnosis and management of bladder cancer: © NICE. Bladder cancer: diagnosis and management of bladder cancer. BJU Int. 2015;2017(120):755–765. 10.1111/bju.14045 29168333

[4] Fankhauser CD, Mostafid H. Prevention of bladder cancer incidence and recurrence: nutrition and lifestyle. Curr Opin Urol. 2018;28(1):88–92. 29211694 10.1097/MOU.0000000000000452

[5] Dong L, Zieren RC, Wang Y, et al. Recent advances in extracellular vesicle research for urological cancers: from technology to application. Biochim Biophys Acta Rev Cancer. 2019;1871:342–360. 30738098 10.1016/j.bbcan.2019.01.008

[6] Marqueen KE, Waingankar N, Sfakianos JP, et al. Early mortality in patients with muscle-invasive bladder cancer undergoing cystectomy in the United States. JNCI Cancer Spectr. 2018;2. pky075. 10.1093/jncics/pky075 PMC6349610 30734024

[7] Wang H, Niu X, Mao F, et al. Hsa_circRNA_100146 Acts as a Sponge of miR-149-5p in Promoting Bladder Cancer Progression via Regulating RNF2. Onco Targets Ther. 2020;13:11007–11017. 33149615 10.2147/OTT.S273622 PMC7605652

[8] Dong W, Bi J, Liu H, et al. Circular RNA ACVR2A suppresses bladder cancer cells proliferation and metastasis through miR-626/EYA4 axis. Mol Cancer. 2019;18(1):95. 31101108 10.1186/s12943-019-1025-z PMC6524247

[9] Wang JX, Liu Y, Jia XJ, et al. Upregulation of circFLNA contributes to laryngeal squamous cell carcinoma migration by circFLNA-miR-486-3p-FLNA axis. Cancer Cell Int. 2019;19:196. 31384171 10.1186/s12935-019-0924-9 PMC6664525

[10] Qu J, Yang J, Chen M, et al. CircFLNA ACTS AS A SPONGE of miR-646 to facilitate the proliferation, metastasis, glycolysis, and apoptosis inhibition of gastric cancer by targeting PFKFB2. Cancer Manag Res. 2020;12:8093–8103. 32982406 10.2147/CMAR.S264674 PMC7490063

[11] Ma C, Wang X, Yang F, et al. Circular RNA hsa_circ_0004872 inhibits gastric cancer progression via the miR-224/Smad4/ADAR1 successive regulatory circuit. Mol Cancer. 2020;19(1):157. 33172486 10.1186/s12943-020-01268-5 PMC7654041

[12] Jemal, A, Siegel, R, Ward, E, Murray, T, Xu, J, and Thun, MJ, et al. Cancer statistics, 2007. CA Cancer J Clin. 2007;57(1):43–66. 10.3322/canjclin.57.1.43 17237035

[13] Stenzl A, Cowan NC, De Santis M, et al. Treatment of muscle-invasive and metastatic bladder cancer: update of the EAU guidelines. Eur Urol. 2011;59(6):1009–1018. 21454009 10.1016/j.eururo.2011.03.023

[14] Travis LB, Curtis RE, Glimelius B, et al. Bladder and kidney cancer following cyclophosphamide therapy for non-Hodgkin’s lymphoma. J Natl Cancer Inst. 1995;87:524–530. 7707439 10.1093/jnci/87.7.524

[15] Czene K, Lichtenstein P, Hemminki K. Environmental and heritable causes of cancer among 9.6 million individuals in the Swedish family-cancer database. Int J Cancer. 2002;99:260–266. 11979442 10.1002/ijc.10332

[16] Lu Q, Liu T, Feng H, et al. Circular RNA circSLC8A1 acts as a sponge of miR-130b/miR-494 in suppressing bladder cancer progression via regulating PTEN. Mol Cancer. 2019;18(1):111. 31228937 10.1186/s12943-019-1040-0 PMC6588875

[17] Jeck WR, Sorrentino JA, Wang K, et al. Circular RNAs are abundant, conserved, and associated with ALU repeats. Rna. 2013;19(2):141–157. 23249747 10.1261/rna.035667.112 PMC3543092

[18] Meng S, Zhou H, Feng Z, et al. CircRNA: functions and properties of a novel potential biomarker for cancer. Mol Cancer. 2017;16(1):94. 28535767 10.1186/s12943-017-0663-2 PMC5440908

[19] Sun Y, Ma G, Xiang H, et al. circFLNA promotes glioblastoma proliferation and invasion by negatively regulating miR‑199‑3p expression. Mol Med Rep. 2021;24(5):786. 34498720 10.3892/mmr.2021.12426 PMC8441964

[20] Pan J, Huang G, Yin Z, et al. Circular RNA FLNA acts as a sponge of miR-486-3p in promoting lung cancer progression via regulating XRCC1 and CYP1A1. Cancer Gene Ther. 2021. DOI:10.1038/s41417-021-00293-w. PMC8761575 33500536

[21] Chen X, Han H, Li Y, et al. Upregulation of long noncoding RNA HOTTIP promotes metastasis of esophageal squamous cell carcinoma via induction of EMT. Oncotarget. 2016;7:84480–84485. 27806322 10.18632/oncotarget.12995 PMC5356674

[22] Yousef EM, Tahir MR, St-Pierre Y, et al. MMP-9 expression varies according to molecular subtypes of breast cancer. BMC Cancer. 2014;14(1):609. 25151367 10.1186/1471-2407-14-609 PMC4150970

[23] Hou Q, Han S, Yang L, et al. The Interplay of MicroRNA-34a, LGR4, EMT-associated factors, and MMP2 in regulating uveal melanoma cells. Invest Ophthalmol Vis Sci. 2019;60(13):4503–4510. 31661551 10.1167/iovs.18-26477

[24] Ayob AZ, Ramasamy TS. Cancer stem cells as key drivers of tumour progression. J Biomed Sci. 2018;25(1):20. 29506506 10.1186/s12929-018-0426-4 PMC5838954

[25] López-Lázaro M. The stem cell division theory of cancer. Crit Rev Oncol Hematol. 2018;123:95–113. 29482784 10.1016/j.critrevonc.2018.01.010

[26] Mitra T, Prasad P, Mukherjee P, et al. Stemness and chemoresistance are imparted to the OC cells through TGFβ1 driven EMT. J Cell Biochem. 2018;119(7):5775–5787. 29537103 10.1002/jcb.26753

[27] Zhang Q, Zhuang J, Deng Y, et al. miR34a/GOLPH3 axis abrogates urothelial bladder cancer chemoresistance via reduced cancer stemness. Theranostics. 2017;7(19):4777–4790. 29187903 10.7150/thno.21713 PMC5706099

[28] Aghaalikhani N, Rashtchizadeh N, Shadpour P, et al. Cancer stem cells as a therapeutic target in bladder cancer. J Cell Physiol. 2019;234(4):3197–3206. 30471107 10.1002/jcp.26916

[29] Zhu F, Qian W, Zhang H, et al. SOX2 is a marker for stem-like tumor cells in bladder cancer. Stem Cell Reports. 2017;9(2):429–437. 28793245 10.1016/j.stemcr.2017.07.004 PMC5550032

[30] Buanne P, Corrente G, Micheli L, et al. Cloning of PC3B, a novel member of the PC3/BTG/TOB family of growth inhibitory genes, highly expressed in the olfactory epithelium. Genomics. 2000;68(3):253–263. 10995567 10.1006/geno.2000.6288

[31] Mao B, Zhang Z, Wang G. BTG2: a rising star of tumor suppressors (review). Int J Oncol. 2015;46(2):459–464. 25405282 10.3892/ijo.2014.2765

[32] Takahashi F, Chiba N, Tajima K, et al. Breast tumor progression induced by loss of BTG2 expression is inhibited by targeted therapy with the ErbB/HER inhibitor lapatinib. Oncogene. 2011;30(27):3084–3095. 21339742 10.1038/onc.2011.24

[33] Tsui KH, Chiang KC, Lin YH, et al. BTG2 is a tumor suppressor gene upregulated by p53 and PTEN in human bladder carcinoma cells. Cancer Med. 2018;7:184–195. 29239139 10.1002/cam4.1263 PMC5773943

[34] Devanand P, Kim SI, Choi YW, et al. Inhibition of bladder cancer invasion by Sp1-mediated BTG2 expression via inhibition of DNA methyltransferase 1. FEBS J. 2014;281:5581–5601. 25284287 10.1111/febs.13099

